# Association between VEGFR-3 expression and lymph node metastasis in non-small-cell lung cancer

**DOI:** 10.3892/etm.2014.2091

**Published:** 2014-11-26

**Authors:** JIE LI, HAN YI, ZHIDONG LIU, HAIQING ZHANG, DEZONG ZHANG, WENTAO YUE, HONGYAN JIA, SHAOFA XU, BAOLAN LI

**Affiliations:** Department of Medical Oncology, Beijing Chest Hospital, Capital Medical University, Tongzhou, Beijing 101149, P.R. China

**Keywords:** non-small cell lung cancer, vascular endothelial growth factor receptor-3, microlymphatic vessel density, semiquantitative multiplex reverse transcription polymerase chain reaction, immunohistochemical

## Abstract

Vascular endothelial growth factor receptor (VEGFR)-3 is considered to be associated with lymphangiogenesis. The aim of the present study was to identify the clinical significance of VEGFR-3 expression and lymph node metastasis in patients with non-small-cell lung cancer (NSCLC). Lung tumor tissue samples and 196 lymph nodes from 52 patients with NSCLC were analyzed. In addition, lung tissue samples and 8 lymph nodes from 10 patients with lung diseases other than cancer were included as controls. Semiquantitative multiplex reverse transcription technology was applied to measure the mRNA expression levels of VEGFR-3, while VEGFR-3 protein expression levels were assessed immunohistochemically. The total number of lymphatic vessels was counted and the microlymphatic vessel density (MLVD) was calculated. The results indicated that the VEGFR-3 mRNA expression level in lymph node tissue from the group with lymph node metastasis was significantly lower compared with the group without lymph node metastasis (0.281±0.166 vs. 0.158±0.158; t=4.849; P<0.001). The VEGFR-3 mRNA expression levels in the lung tumor tissue of the NSCLC patients exhibited no statistically significant difference between the lymph node metastasis and lymph node non-metastasis groups (0.139±0.137 vs. 0.142±0.123; t=0.08; P>0.05). In addition, in the lymph node metastasis group, there was no statistically significant difference between the metastasis-positive and -negative lymph nodes (0.158±0.158 vs. 0.123±0.115; t=0.993; P>0.05) with regard to VEGFR-3 mRNA expression. Morphologically, VEGFR-3 immunoreactivity was primarily localized in the cytoplasm of the lymphatic endothelial cells, as well as a number of the cancer cells. MLVD was much higher in the lung tissue surrounding the tumor than in the tumor tissue, and was significantly higher in the lymph node metastasis group than in the lymph node non-metastasis group. VEGFR-3 expression levels were shown to correlate with lymph node metastasis in NSCLC patients, thus, may be a useful biomarker for lymph node metastasis prediction in NSCLC. MLVD is a key indictor of lymphatic vessel metastasis in NSCLC. An enhanced MLVD indicates lymphangiogenesis and lymphatic node metastasis, and may be an important predictor for tumor monitoring and prognosis.

## Introduction

Invasion and metastasis are the main characteristics during the progression of malignant tumors, which is responsible for the majority of cancer mortalities. Tumor dissemination may occur through a number of pathways, among which blood vessels and lymphatics are key components of metastatic spread. Lymph node metastasis is an important prognostic indicator in a number of cancer types. Numerous epithelial tumors have been characterized by lymph node metastasis, which is often an early event in tumor progression. Lymphatic metastasis is also a key factor associated with tumor recurrence and prognosis. Previous studies on tumor molecular biology have revealed that the development of a microvascular network (angiogenesis and lymphangiogenesis) is essential for tumor metastasis.

Vascular endothelial growth factor receptor (VEGFR)-3 was the first cloned lymphatic marker, and is predominantly expressed on lymphatic endothelium in adult tissues. On binding to its ligands, VEGF-C and VEGF-D, VEGFR-3 signals for tumor lymphangiogenesis, mediating tumor metastasis to the lymph nodes (1,2). Therefore, the inhibition of lymphangiogenesis is a realistic therapeutic strategy for inhibiting tumor cell dissemination and lymphatic metastasis. Previous studies have predominantly focused on tumor metastasis via the blood vasculature and significant progression has been made with regard to angiogenesis and antiangiogenesis therapy (3). However, antiangiogenesis therapy appears to be not as efficient as predicted for the treatment of tumor metastasis, which may be due to the networking of the blood and lymphatic vasculatures. Blocking a single route of metastasis is unable to inhibit the distant metastasis of tumor cells, which may transfer between the two vasculatures (4).

In the present study, mRNA and protein expression levels of VEGFR-3 were detected in non-small-cell lung carcinoma (NSCLC) tissues and lymph nodes using semiquantitative reverse transcription polymerase chain reaction (RT-PCR) and immunohistochemisty. In addition, the microlymphatic vessel density (MLVD) was calculated in order to analyze the correlation with lymph node metastasis, which may be an indicator of tumor metastasis and provide evidence for personalized therapy.

## Materials and methods

### Study criteria

In total, 52 patients who had been diagnosed with primary NSCLC in Beijing Chest Hospital (Beijing, China) between April 2006 and June 2007 were selected for the study. The patients had not undergone any previous treatment and were aged between 29 and 77 years (mean age, 59±11 years). In total, 38 patients were male, while 14 patients were female. The histological types of the lung cancer tissues were classified into adenocarcinoma (20 cases), squamous cell carcinoma (27 cases) and adenosquamous carcinoma (5 cases), according to the World Health Organization’s standards (5). All the patients provided informed consent. According to the postoperative pathology results, patients with at least one lymph node metastasis were classified as the lymph node metastasis-positive group (25 patients), while patients without lymph node metastasis were classified into the lymph node metastasis-negative group (27 patients). In total, 196 lymph nodes were analyzed, including 72 metastasis-positive lymph nodes and 26 metastasis-negative lymph nodes from the lymph node metastasis-positive group and 98 lymph nodes from the lymph node metastasis-negative group. In the group of patients with benign lung disease, 10 lung tissues and 8 lymph nodes were analyzed. The study was approved by the Ethics Committee of Beijing Chest Hospital (Beijing, China).

### Reagents

TRIzol was purchased from Invitrogen Life Technologies (Carslbad, CA, USA). PCR primers were synthesized by Shanghai Shengwu Gongcheng Co., Ltd. (Shanghai, China), while dNTPs and Moloney murine leukemia virus (M-MLV) reverse transcriptase were obtained from Promega Corporation (Madison, WI, USA). RNasin Ribonuclease Inhibitor was purchased from Huamei, while rabbit anti-VEGFR-3 antibodies and an SP-9000 ELISA kit were purchased from Zhongshan Jinqiao Biotechnology, Co., Ltd. (Zhongshan, China).

### RT-PCR of VEGFR-3

Tissue samples were collected within 30 min following surgery and were stored in liquid nitrogen immediately.

For RNA extraction, the tissues were ground in liquid nitrogen and the RNA was extracted using TRIzol and chloroform. The optical density at 260 and 280 nm was detected using a UV-spectrophotometer (Shimadzu Corporation, Kyoto, Japan), with a spectral bandwidth of 1.8–2.0. The RNA samples were also analyzed by formaldehyde denaturing gel electrophoresis and no degradation was detected. RNA samples were diluted to 1 μg/μl and stored at −80°C.

For reverse transcription, 2 μg RNA template, 1 μl oligo(dT) and RNase free H_2_O were placed in a microcentrifuge tube to a final volume of 15 μl and incubated at 70°C for 5 min. The samples were centrifuged at 300 × g, 4°C for 30 sec in a microcentrifuge and then placed on ice. A 25 μl reaction was prepared by adding the following reagents in the order listed: 5 μl 5X first strand buffer, 25 units RNasin RNase inhibitor, 200 units M-MLV reverse transcriptase, 5 μl 4X dNTP and nuclease-free water. The reaction mixture was incubated at 42°C for 60 min. The samples were then heated at 95°C for 5 min to inactivate the reverse transcriptase and incubated at 0–5°C for 5 min.

The primers were designed according to the DNA sequence of VEGFR-3 (6). The primer sequences were as follows: VEGFR-3 upstream, CCCACGCAGACATCAAGACG and downstream, TGCAGAACTCCACGATCACC; β-actin upstream, TGACGGGGTCACCCACACTGTGCCCATCT and downstream, CTAGAAGCATTTGCGGTGGACGAT GGAGGG.

The following conditions were selected for the PCR reaction: Predenaturation at 94°C for 5 min; denaturation at 94°C for 30 sec; annealing at 60°C for 1 min; extension at 72°C for 1 min; for 32 cycles. The final extension was conducted at 72°C for 10 min. A reaction without a template was used as the negative control and the PCR products were separated by 1.5% agarose gel electrophoresis (Invitrogen Life Technologies, Carlsbad, CA, USA).

### Calculating the VEGFR-3 protein expression levels in the clinical samples and lymphatic vessels

The study consisted of 52 lung tumor tissues collected from NSCLC patients. Formalin-fixed lung tumor tissue samples were embedded in paraffin and cut into 4-μm slices. The sections were detected using an immunohistochemical streptavidin-biotin complex kit (Beijing Solarbio Science and Technology, Beijing, China). According to the manufacturer’s instructions, the slides were restored using citric acid buffer (pH 6.0) under high pressure conditions. Immunohistochemical staining was performed to analyze the expression levels of VEGFR-3 using specific antibodies, which was followed by staining with horseradish peroxidase-conjugated secondary antibodies. Next, the slides were developed in diaminobenzidine and counterstained with hematoxylin. The stained slides were dehydrated and mounted in permount solution and visualized using a microscope.

### Immunohistochemical analysis

VEGFR-3 expression was primarily localized in the cytoplasm. The results were assessed using a semiquantitative scoring method, with the positive staining score standard as follows: 0 was no color (same as the background color); 1 was pale yellow (slightly higher than the background color); 2 was brown (significantly higher than the background color); and 3 was strong brown. In total, 400 tumor cells were selected at a high magnification for scoring according to the percentage of positive cells: 0, negative; 1, <10%; 2, 11–50%; 3, 51–75%; and 4, >75%. Positive immunohistochemical results were assessed by the product of the positive staining score and the positive cell score: 0–2*,* negative (−); 3–4, weak positive (+); 5–8, moderate positive (++); and 9–12, strong positive (+++). Overall, 4–12 was considered to be positive, while 0–3 was considered to be negative (7).

### MLVD assay

The VEGFR-3 positive microlymphatic vessel area (hot zone) was identified and the MLVD was counted in five high power fields (HPFs). The mean value of the HPF was the MLVD of the tissue. A single or cluster of endothelial cells was selected for a vessel count. The microlymphatic vessels associated with the muscular layer were not selected for counting (7).

### Statistical analysis

SPSS software (version 13.0; SPSS, Inc., Chicago, IL, USA) was used for statistical analysis. The VEGFR-3 mRNA expression levels are expressed as the mean ± standard deviation and comparisons between groups were conducted using the Student’s t test. Immunohistochemical data were analyzed with a χ^2^ test. P<0.05 was considered to indicate a statistically significant difference.

## Results

### Semiquantitative RT-PCR

In the lung tumor tissue, the mRNA expression levels of VEGFR-3 were significantly higher than in the benign tissues (0.140±0.129 vs. 0.031±0.043; t=2.598; P<0.05). In addition, the mRNA expression levels of VEGFR-3 in the lung tumor tissue with positive and negative lymph node metastasis exhibited no statistically significant difference (0.139±0.137 vs. 0.142±0.123; t=0.08; P>0.05).

The mRNA expression levels of VEGFR-3 in the lymph nodes (98 samples) from the lymph node metastasis-negative group and metastasis-positive lymph nodes (72 samples) from the lymph node metastasis-positive group exhibited statistically significant differences (0.281±0.166 vs. 0.158±0.158; t=4.849, P<0.001; Table I). The mRNA expression of VEGFR-3 in the metastasis-positive (72 cases) and metastasis-negative lymph nodes (26 cases) from the lymph node metastasis-positive group exhibited no statistically significant difference (0.158±0.158 vs. 0.123±0.115; t=0.993; P>0.05 Table II).

### Location of VEGFR-3

**Figure 1:**
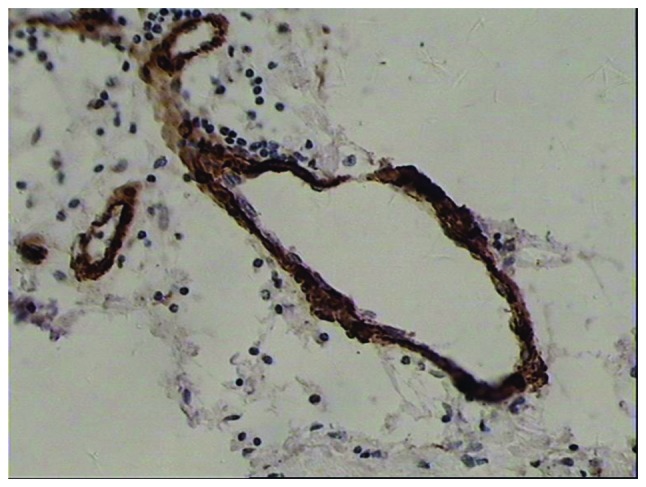
Large lymphatic vessels were observed in the peritumoral tissue with irregular vascular walls and VEGFR-3 positive staining (magnification, ×100). A horseradish peroxidase-conjugated secondary antibody were used, and hematoxylin was used as a counterstain for the HRP-conjugated secondary antibody. VEGFR, vascular endothelial growth factor receptor.

**Figure 2:**
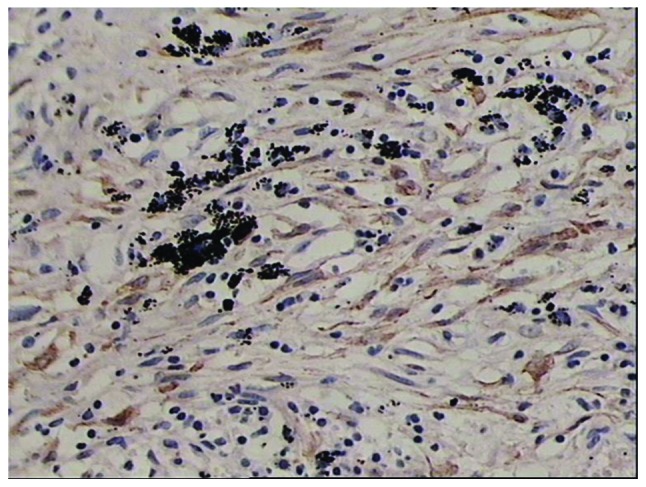
Lymphatic capillary network in the peritumoral tissue (magnification, ×100). A horseradish peroxidase-conjugated secondary antibody were used, and hematoxylin was used as a counterstain for the HRP-conjugated secondary antibody.

**Figure 3:**
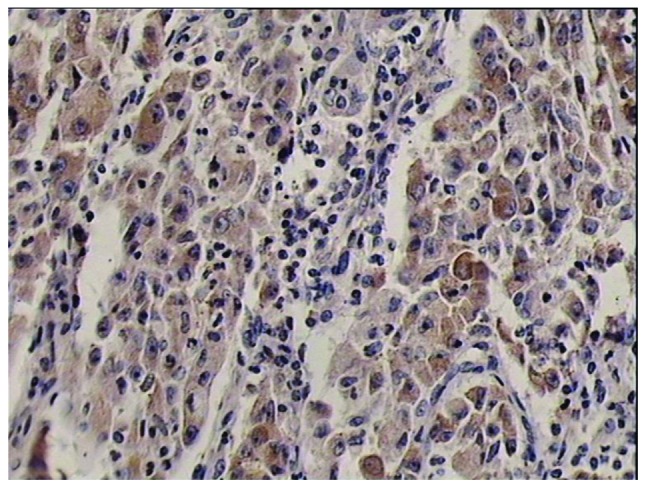
VEGFR-3 positive tumor cells in lung cancer tissue (magnification, ×200). A horseradish peroxidase-conjugated secondary antibody were used, and hematoxylin was used as a counterstain for the HRP-conjugated secondary antibody. VEGFR, vascular endothelial growth factor receptor.

**Figure 4:**
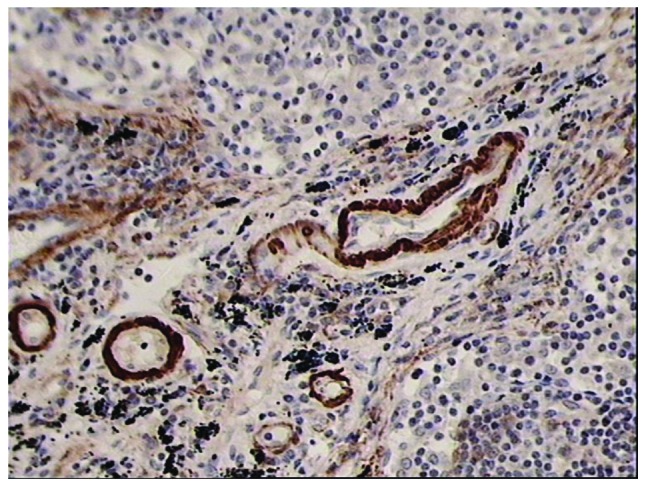
VEGFR-3 positive vessels were observed in the lymph nodes. The irregular vessels are lymphatic, while the round tubes are small vessels (magnification, ×100). A horseradish peroxidase-conjugated secondary antibody were used, and hematoxylin was used as a counterstain for the HRP-conjugated secondary antibody. VEGFR, vascular endothelial growth factor receptor.

**Figure 5:**
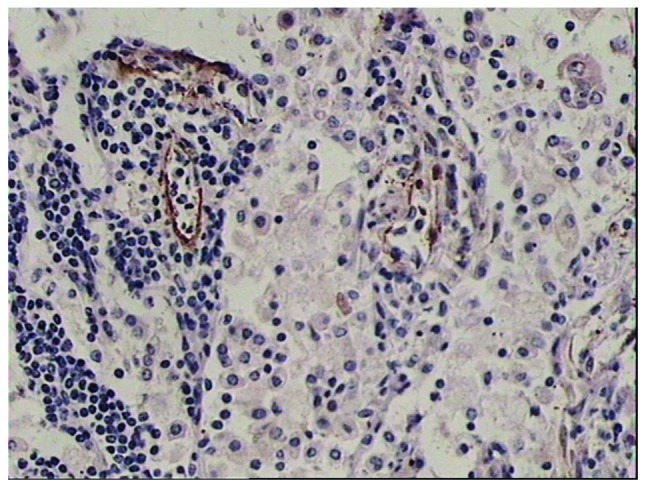
VEGFR-3 positive, small lymphatic vessels were observed in the peritumoral tissue with thin walls, discontinuous endothelial cells and interruptions of lymphocytes (magnification, ×100). A horseradish peroxidase-conjugated secondary antibody were used, and hematoxylin was used as a counterstain for the HRP-conjugated secondary antibody. VEGFR, vascular endothelial growth factor receptor.

Positive staining of VEGFR-3 was indicated by brown staining and was observed in peritumoral and intratumoral lymphatic endothelial cells and part of the cancer cell plasma. No expression was observed in the adjacent normal bronchi and alveoli (Figs. 1–5).

### Association between VEGFR-3 expression and lymph node metastasis

VEGFR-3 expression levels were positive in 27 of 52 cases (51.9%) of NSCLC tissue, while positive expression was only observed in one case (10%) in the control group. Statistically significant differences were observed between the groups (χ^2^=5.856; P<0.05). The expression of VEGFR-3 in patients with lymph node metastasis (72% positive) was also significantly higher than those without lymph node metastasis (33.3% positive).

### VEGFR-3 positive vessel count

Under a magnification of ×200, the number of VEGFR-3 positive vessels in the tumor and peritumoral tissues exhibited statistically significant differences (9.88±3.22 vs. 3.40±1.27; t=22.125; P<0.05). The number of VEGFR-3 positive vessels in the peritumoral tissues of the lymph node metastasis-positive and -negative groups also exhibited a statistically significant difference (12.72±1.86 vs. 7.26±1.51; t=11.665; P<0.05). In addition, the number of VEGFR-3 positive tubes was shown to correlate with VEGFR-3 protein expression (Table III). Thus, VEGFR-3 expression is associated with lymph node metastasis (Table IV).

## Discussion

Lung cancer is one of the most common malignant tumors, causing a severe threat to human health. Lymph node metastasis of lung cancer directly influences clinical treatment and patient prognosis. The survival rates for patients with lymph node metastasis are significantly lower than those for patients without lymph node metastasis. With the identification of lymphangiogenesis factors (VEGF-C and VEGF-D) and lymphatic endothelial cell specific markers, including VEGFR-3, lymphatic vessel endothelial hyaluronan receptor-1 and prospero homeobox protein-1, lymphangiogenesis and lymphatic metastasis have been increasingly studied.

The first cloned molecular marker of lymphatic endothelial cells was VEGFR-3, the gene of which is located on 5q33–q35. VEGFR-3 is essential for the initial formation of the cardiovascular network prior to the onset of the lymphatic system. In the early phase of embryonic development, VEGFR-3 is expressed on developing blood endothelial cells and is required for normal vascular development. In later development, VEGFR-3 is specifically expressed on lymphatic vessels and regulates occurrence and growth. However, in a number of pathological conditions, including cancer and inflammation, VEGFR-3 has been shown to be expressed in the endothelium of the microvasculature (8–11).

At present, whether functional tumor lymphatic vessels exist remains controversial. In the present study, immunohistochemical analysis of 52 lung cancer patients demonstrated that VEGFR-3 is expressed not only in lymphatic endothelial cells, but also in microvascular endothelial cells and the tumor cell cytoplasm, which is in accordance with the studies by Li *et al* and Peng *et al* (12,13). Newly formed lymphatic capillaries in NSCLC tissues undergo expansion, with thin walls, no significant plasma membrane protrusions or continuous endothelial cells and in certain cases, without endothelial cell coverage. Peritumoral tissues have a higher number of newly formed lymphatic capillaries, exhibiting cord-like structures with small branches and thin walls. A number of the capillaries have a very small cavity and only appear as a brown mass with light microscopy.

Although the expression of VEGFR-3 has been studied extensively in a variety of tumor tissues, to the best of our knowledge, there are no studies investigating the expression in lymph nodes. The present study detected the expression levels of VEGFR-3 in the lung tissue and lymph nodes using semiquantitative RT-PCR and immunohistochemistry. The results not only confirmed the association between VEGFR-3 and tumor lymphangiogenesis and lymph node metastasis, but also demonstrated that high VEGFR-3 expression levels in metastatic-negative lymph nodes can be an indicator of lymph node metastasis.

In the present study, VEGFR-3 positive lymphatic vessels were analyzed. With immunohistochemical staining, VEGFR-3 positive lymphatic vessels were found to predominantly exist in the peritumoral tissue, particularly in the region between the tumor and normal tissue, which is also referred to as the tumor-infiltrating area. However, VEGFR-3 positive lymphatic vessels were rarely observed within the tumor area. The number of VEGFR-3 positive lymphatic vessels in the peritumoral tissue was significantly higher compared with the tumor tissue. In addition, the expression levels were higher in the NSCLC tissue with lymph node metastasis as compared with the tissue without lymph node metastasis (P<0.05), indicating that tumor lymphangiogenesis is associated with tumor invasion. As the proportion of intratumoral lymphatic vessels was small, we hypothesized that peritumoral lymphatic vessels play a more important role in the invasion and metastasis of lung cancer. The decreasing number of VEGFR-3 positive lymphatic vessels in tumors may be associated with the following factors. Firstly, the lack of an original structure of the lymphatic valves within the tumor may prevent the intake of tissue fluid and suppress lymphatic functions. Secondly, continuous growth of tumor cells produces mechanical force, which causes lymphatic atrophy and non-function. Finally, tumor cells invade and destruct the lymphatic network, thus, leave only lymphatic vessel epithelial remnants within the tumor. With regard to the increase in VEGFR-3 lymphatic vessels in the peritumoral tissue, we hypothesized that tumor cells secreting VEGF-C induced the proliferation of peritumoral lymphatic vessels via VEGFR-3, which is located on the lymphatic endothelium, including increasing the diameter and the number of peritumoral lymphatic vessels. Tumor cells maintain close contact with the lymphatic endothelium, then infiltrate into the lymphatic vessels and move to the regional lymph nodes, proliferating there and finally resulting in lymphatic metastasis (1).

In the present study, VEGFR-3 mRNA expression in the lung cancer tissues exhibited no significant correlation with lymph node metastasis. A possible reason may be that expression of VEGFR-3 in the cytoplasm of tumor cells and small blood vessels may not be detected by RT-PCR. Therefore, immunohistochemistry and the MLVD counting method may be more suitable for investigating VEGFR-3 expression. The association between the expression of VEGFR-3 in lung cancer tissues and lymph node metastasis remains controversial (14,15), and the exact mechanism requires further investigation.

The mRNA expression levels of VEGFR-3 were also detected in the lymph node tissue. The VEGFR-3 mRNA expression level in the lymph node tissue from the lymph node metastasis group was significantly lower than that from the group without lymph node metastasis (P<0.05; 0.158±0.158 vs. 0.281±0.166; P<0.001).

However, no statistically significant difference was observed in the VEGFR-3 mRNA expression level between the metastasis -positive and -negative lymph nodes from the NSCLC patients with lymph node metastasis (P>0.05). The result was consistent with a previous study investigating VEGF-C mRNA expression levels in lymph nodes (16), indicating that pathologically negative lymph node tissue may exhibit metastasis at a molecular level.

The present study applied semiquantitative RT-PCR and immunohistochemisty to investigate the association between VEGFR-3 mRNA expression and lymph node metastasis in NSCLC patients. However, the conclusions obtained by these two methods were not consistent since VEGFR-3 expression in the cytoplasm of tumor cells and small blood vessels can not be detected using RT-PCR methods. Thus, we hypothesize that VEGFR-3 should be used as a marker of lymphatic endothelial cells to assess the number of lymphatic vessels, which correlates with lymph node metastasis of lung cancer. For this purpose, the immunohistochemistry method was more appropriate compared with the semiquantitative RT-PCR method.

## Figures and Tables

**Table I:** Comparison of VEGFR-3 mRNA expression levels between metastasis-positive lymph nodes and lymph nodes from lung cancer patients without metastasis.

Classification	Cases, n	VEGFR-3	t-value	P-value
Negative LN	98	0.281±0.166	4.849	0.000
Positive LN	72	0.158±0.158		

VEGFR, vascular endothelial growth factor receptor; LN, lymph node.

**Table II:** Comparison of VEGFR-3 mRNA expression levels between negative and positive lymph nodes from lung cancer patients with LN metastasis.

Classification	Cases, n	VEGFR-3	t-value	P-value
Negative LN	26	0.123±0.115	0.993	0.323
Positive LN	72	0.158±0.158		

VEGFR, vascular endothelial growth factor receptor; LN, lymph node.

**Table III:** Association between VEGFR-3 positive vessels and the expression of VEGFR-3 in tumor tissue.

Classification	Vessels, n	Peritumoral MLVD	t-value	P-value
VEGFR-3 (−)	25	8.040±2.525	4.734	0.000
Protein expression (+)	27	11.593±2.859		

VEGFR, vascular endothelial growth factor receptor; MLVD, microlymphatic vessel density.

**Table IV:** Association between VEGFR-3 expression in tumor tissues and lymph node metastasis.

	VEGFR-3 expression			
			
Classification	(−)	(+)	χ^2^	P-value
Lymph node (−)	18	9	7.775	0.005
Metastasis (+)	7	18		

VEGFR, vascular endothelial growth factor receptor.
